# One‐pot dual protein labeling for simultaneous mechanical and fluorescent readouts in optical tweezers

**DOI:** 10.1002/pro.70098

**Published:** 2025-03-18

**Authors:** Laura‐Marie Silbermann, Maximilian Fottner, Ronald van der Meulen, Nora Migdad, Kathrin Lang, Katarzyna Tych

**Affiliations:** ^1^ Groningen Biomolecular Sciences and Biotechnology Institute University of Groningen Groningen the Netherlands; ^2^ Laboratory for Organic Chemistry (LOC), Department of Chemistry and Applied Biosciences (D‐CHAB) ETH Zurich Zurich Switzerland

**Keywords:** bioconjugation, chaperone protein 90, non‐canonical amino acid, optical tweezers, protein dynamics, single‐molecule FRET

## Abstract

Optical tweezers are widely used in the study of biological macromolecules but are limited by their one‐directional probing capability, potentially missing critical conformational changes. Combining fluorescence microscopy with optical tweezers, employing Förster resonance energy transfer (FRET) pairs, addresses this issue. When integrating fluorescence microscopy with optical tweezers, orthogonal protein conjugation methods are needed to enable simultaneous, site‐specific attachment of fluorophores and DNA handles, commonly used to apply force to molecules of interest. In this study, we utilized commercially available reagents for dual site‐specific labeling of the homodimeric heat shock protein 90 (Hsp90) using thiol‐maleimide and inverse electron demand Diels–Alder cycloaddition (IEDDAC) bioorthogonal reactions. In a one‐pot approach, Hsp90 modified with a cysteine mutation and the non‐canonical amino acid cyclopropene‐L‐lysine (CpK) was labeled with the FRET pair maleimide‐Atto 550 and maleimide‐Atto 647N, alongside single‐stranded methyltetrazine‐modified DNA oligonucleotide. Optical tweezers experiments with this labeled Hsp90 construct revealed structural transitions consistent with previous studies, validating the approach. Fluorescence measurements confirmed the proximity of FRET pairs in the N‐terminally closed state of Hsp90 in this experimental setup. This integrative method provides a powerful tool for probing complex protein conformational dynamics beyond the limitations of traditional optical tweezers.

## 1 INTRODUCTION

A limitation of optical tweezers when applied to the study of biological macromolecules arises from their one‐directional probing of structural changes such as unfolding events and conformational changes. This may cause vital information about the studied molecule to go undetected. To overcome this, strategies like engineering multiple force application points within the same molecule to vary the pulling directions (Ziegler et al. 2016) and using a quadrupole trap (Dame et al. 2006) have been employed. A rapidly growing approach for acquiring comprehensive information about biomolecular systems is the combination of fluorescence microscopy with optical tweezers (Bustamante et al. 2021; Choudhary et al. 2019). By incorporating a Förster resonance energy transfer (FRET) pair into a protein tethered in an optical tweezers setup, it becomes possible to simultaneously detect structural changes that do not occur along the mechanically probed spatial coordinate.

Protein synthesis for simultaneous mechanical and FRET readout in optical tweezers (OT) combined with fluorescence microscopy experiments has previously been achieved using a sophisticated, customized cell‐free protein synthesis system to study protein folding within the ribosomal exit tunnel (Wruck et al. 2021). In contrast, our study presents, to our knowledge, the first use of a much more widely adopted *Escherichia coli*‐based protein synthesis system to generate protein constructs suitable for simultaneous mechanical and FRET readout in OT experiments.

A crucial consideration for this approach is selecting orthogonal protein conjugation methods for simultaneous, site‐specific attachment of fluorophores and DNA handles. DNA handles are commonly used to tether the protein to optically trapped beads, separating the protein from the high‐intensity laser traps to minimize photodamage.

Decisive factors in choosing two orthogonal protein conjugation strategies include biocompatibility, reagent availability, impact on protein activity, cost, reaction efficiency, and compatibility for a one‐pot approach, among others.

Various strategies for attaching DNA handles have been described in recent literature reviews (van der Sleen and Tych 2021; Yang et al. 2020). When targeting a residue within the protein rather than the N‐ or C‐terminus, conjugation strategies involving single amino acid mutations are preferred over short peptide tags to minimize the risk of affecting protein function. Exploiting the low abundance of cysteines in proteins (<2%) and their distinctive thiol group for selective reactions (Spicer and Davis 2014) has become a widely used approach in creating protein‐DNA chimeras for optical tweezers experiments. Methods include the simple and fast approach of thiol‐maleimide coupling (Jahn et al. 2014). However, the range of functional groups present in natural amino acids is limited, and their abundance poses challenges for achieving site specificity. A solution to this limitation is the use of non‐canonical amino acids bearing bioorthogonal handles, whose incorporation into proteins has been extensively optimized in recent years, enabling relatively high yields (Chin 2017; Lang and Chin 2014; Scinto et al. 2021; Young et al. 2010).

With these considerations in mind, we chose to use thiol‐maleimide chemistry to attach the commercially available FRET pair, maleimide‐Atto 550 and maleimide‐Atto 647N (ATTO‐TEC, Germany). Additionally, we employed the inverse electron demand Diels‐Alder cycloaddition (IEDDAC) (Mattheisen et al. 2023; Pagel 2019) for the reaction between the commercially available single‐stranded methyltetrazine‐modified DNA oligonucleotide (e.g., from Biomers, Germany) and the commercially available non‐canonical amino acid cyclopropene‐L‐lysine (CpK) (Elliott et al. 2014) (e.g., from SiChem GmbH, Germany). This non‐canonical amino acid was incorporated into the Hsp90 protein from *Saccharomyces cerevisiae* (Hsp82).

## 2 RESULTS

### 2.1 Generating Hsp90 protein with two site‐specific chemical attachment sites

**FIGURE 1:**
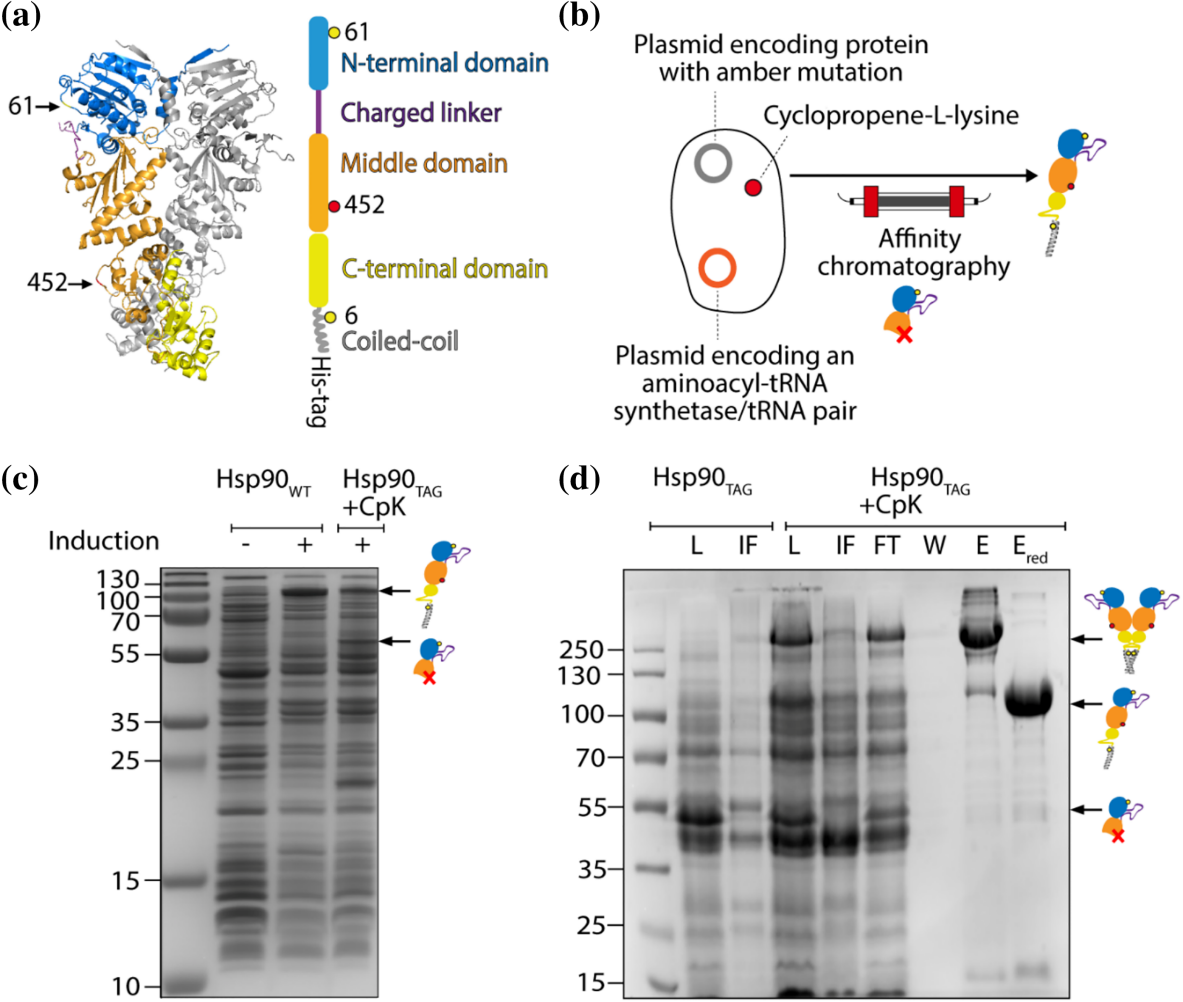
Producing Hsp90 protein featuring two site‐specific chemical attachment sites for labeling. (a) The crystal structure (PDB accession code: 2CG9) is shown with one monomer colored by its different structural elements and the positions of the mutations shown: the NTD (blue), charged linker—not fully resolved in the crystal structure—(purple), MD (orange), C‐terminal domain (CTD, yellow), coiled‐coil (gray), position 61 in the NTD (yellow), and position 452 in the MD (red). Introduced modifications are cysteines at position 61 (D61C) and 6 (A6C) (yellow circles) as well as an amber stop codon at position 452 (D452TAG, red circle). (b) Expression was performed using *E. coli* K12 cells harboring two plasmids: one with the Hsp90 construct, and the other plasmid with an aminoacyl‐tRNA synthetase (aaRS)/tRNA pair that directs the incorporation of CpK in response to the amber codon. Truncated protein resulting from incomplete amber suppression was removed by affinity chromatography using a C‐terminal affinity tag. (c) SDS‐PAGE gel of cell lysate supernatant from Hsp90_WT_ and Hsp90‐D452TAG under various expression conditions. (d) SDS‐PAGE gel of samples from Ni‐IMAC purification include: load (L), flow through (FT), insoluble fraction (IF), wash (W), eluate (E), and eluate with reducing agent (E_red_).

Although Hsp90 remains enigmatic in many ways (Silbermann et al. 2024), it has already been extensively studied in optical tweezers experiments (Jahn et al. 2014; Jahn et al. 2016; Jahn et al. 2018; Tych et al. 2018) making it a suitable model protein for developing an optical tweezers method involving a tethered, fluorescently labeled protein. To allow for simultaneous, site‐specific labeling of Hsp90 with commercially available fluorescent dyes and short single‐stranded DNA oligonucleotides, we inserted two chemical attachment sites. This was achieved by site‐directed mutagenesis. The amino acid sequence of the construct is provided in Data S1, Supporting Information. One attachment site was introduced by creating a cysteine mutation in the N‐terminal domain (NTD) at amino acid position 61, and the other by introducing an amber stop codon at amino acid position 452 in the middle domain (MD) for incorporation of the non‐canonical amino acid CpK via genetic code expansion (Figure 1a,b). Both amino acid positions, 61 and 452, are surface‐exposed according to a published crystal structure in the PDB database (PDB accession code: 2CG9). For more detailed information on assessing the accessibility of an amino acid position for labeling from the protein crystal structure, we refer the reader to Gross and Lehman (2013). The C‐terminal coiled‐coil motif, which has been previously reported and utilized, stabilizes the dimeric form of Hsp90. The cysteines in the coiled‐coil motif are closely spaced, promoting disulfide bond formation, covalently linking the monomers and facilitating re‐dimerization during repeated pulling and relaxation cycles in optical tweezers experiments. Cysteines at position 61 are further apart and less likely to be involved in disulfide bond formation (Jahn et al. 2016). Positions 61 in dimeric Hsp90 are suitable for labeling with a FRET pair, as previous single‐molecule FRET experiments (Hellenkamp et al. 2017) revealed that these positions are in close enough proximity in the N‐terminally closed state (87.8 Å) but not in the N‐terminally open state ensemble for FRET to occur. Interdomain fluctuations of up to 25 Å have been reported for the ensemble of open state conformations in the absence of an applied force.

**FIGURE 2:**
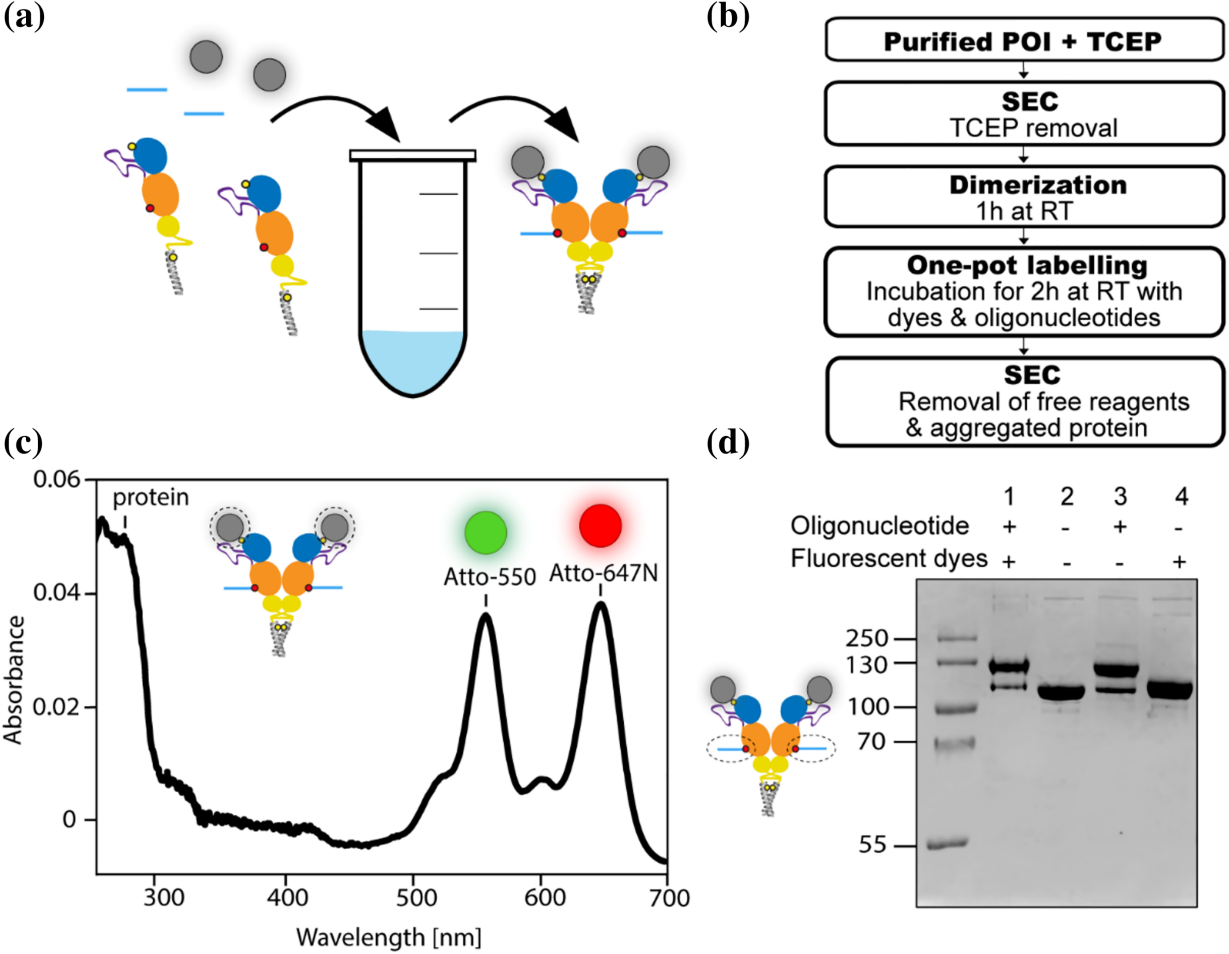
Labeling with DNA oligonucleotides and a FRET pair using a one‐pot approach. (a) One‐pot functionalization with single‐stranded methyltetrazine‐modified DNA oligonucleotide (blue) via reaction with the non‐canonical amino acid CpK (red), and attachment of the maleimide‐Atto 550 and maleimide‐Atto 647N FRET pair (gray) to cysteine (yellow). (b) Functionalization of the protein and purification scheme. (c) Degree of labeling with fluorescent dyes determined spectrophotometrically. (d) Degree of labeling with DNA oligonucleotide determined by SDS‐PAGE analysis and protein band quantification using ImageJ. The sample used for OT analysis (lane 1) was run together with SEC‐purified non‐labeled Hsp90 (lane 2) and other control samples, as indicated. All samples were run with a reducing agent.

CpK, added to the culture medium of *E. coli* K12 cells co‐transformed with two plasmids—one carrying the modified Hsp90 and the other carrying an orthogonal aminoacyl‐tRNA synthetase (aaRS)/tRNA pair for CpK encoding—was successfully incorporated at position 452 via amber (TAG) stop codon suppression. Amber suppression of Hsp90‐D452TAG with CpK showed good yields when compared to Hsp90_WT_, and only little amounts of truncated protein (52.3 kDa) (Figure 1c). Truncated Hsp90 was successfully removed by Ni‐IMAC affinity chromatography (lanes E and E_red_ in Figure 1d) utilizing the C‐terminal His‐tag on full‐length Hsp90. Additional SEC purification enabled the isolation of protein of high purity (Figure 2d). From a 1 L culture, 1.8 mg of purified protein was obtained, which was subsequently used for labeling with FRET pair dyes and short single‐stranded DNA oligonucleotides.

It is generally essential to check that the biological function and folding of the protein are not impacted by mutagenesis and labeling. To check that the biological function of Hsp90 is not affected by the done modifications, a well‐established activity assay (Hessling et al. 2009; Jahn et al. 2014) is employed. This assay has already been used to demonstrate that the introduction of the artificial coiled‐coil, mutations at positions 61 and 452, as well as labeling of these positions with Atto‐dyes, has no effect on the ATPase activity of yeast Hsp90 (Schmid and Hugel 2020; Sohmen et al. 2023). The measured activity of our construct was determined to be 1.1 min^−1^ (±0.4 min^−1^), which is comparable to results of previous studies for Hsp90_WT_, 1.4 min^−1^ (±0.3 min^−1^) (Sohmen et al. 2023). Additionally, proper folding of yeast Hsp90 after mutagenesis and labeling with DNA‐oligonucleotides has already been demonstrated in multiple studies (Jahn et al. 2014; Mondol et al. 2023; Tych et al. 2018).

### 2.2 Labeling with DNA oligonucleotides and a FRET pair using a one‐pot approach

Labeling was performed using an efficient one‐pot approach (Figure 2a) that combines two mutually orthogonal chemistries. Combining these chemistries in a one‐pot setup has already been successfully demonstrated using a small molecule substrate (Knall et al. 2016). One of the chemistries involves the irreversible and relatively rapid IEDDAC between the non‐canonical amino acid CpK (Nɛ‐(((2‐methylcycloprop‐2‐en‐1‐yl)methoxy)carbonyl)‐L‐lysine) in the Hsp90 MD and single‐stranded methyltetrazine‐modified DNA oligonucleotide. More specifically, a DNA oligonucleotide functionalized with a 3′‐aminolink‐C6 and 2‐(4‐(6‐Methyl‐1,2,4,5‐tetrazin‐3‐yl)phenyl)acetic acid from Biomers, Germany, was used. The other chemistry uses a thiol‐maleimide reaction to label the cysteine at position 61 in the NTD of dimeric Hsp90 with the FRET pair Atto 550 and Atto 647N.

When selecting a suitable FRET pair, there are many factors to consider. For single‐biomolecule applications, fluorescent labels should be small, bright, and long‐lasting (Juette et al. 2014). Therefore, organic dyes are commonly used for these applications (Bustamante et al. 2021). Using more hydrophilic dyes minimizes the risk of non‐specific adhesion via hydrophobic interactions, which can introduce artifacts into the data (Zanetti‐Domingues et al. 2013) and adversely affect protein folding and function (Gust et al. 2014). Common FRET pairs for confocal single‐molecule studies are Cy3‐Cy5, Atto 550‐ATTO 647N, and Alexa 488‐Alexa 647 (Baibakov et al. 2020; Gust et al. 2014; Roy et al. 2008). The Atto 647N dye chosen here is one of the most widely used fluorescent dyes in single‐molecule studies (Gust et al. 2014), thanks to its exceptional brightness and photostability in the near‐infrared spectral region (Vogelsang et al. 2008). Moreover, the FRET pair Atto 550 and Atto 647N has already been successfully used at position 61 of yeast Hsp90 (Hellenkamp et al. 2017). The most suitable choice of dye is defined by the specific experimental design, and we refer the reader to comprehensive reviews for further information on this topic (Gust et al. 2014; Juette et al. 2014; Roy et al. 2008).

The thiol‐maleimide reaction used to attach maleimide‐Atto 550 and maleimide‐Atto 647 requires cysteine reduction prior to adding labeling reagents (Figure 2b). The removal of the reducing agent (here TCEP) before dimerization and labeling was initially attempted using a MiniTrap G‐25 column, known for its higher step yield compared to the larger SEC columns typically used for protein purification. However, using a MiniTrap G‐25 column negatively affected the degree of covalent dimerization, leading to a switch to a Superdex 200 column. After labeling, free oligonucleotides, dyes, and aggregated proteins were removed by SEC.

Subsequently, the degree of labeling with dyes (DOL_dye_) was determined spectrophotometrically (Figure 2c) as described in the Methods section. The maximum achievable proportion of dimeric Hsp90 labeled with a FRET pair is 50%. This value is derived as follows: the two dyes, Atto 550 and Atto 647N, were added to the homodimeric protein simultaneously at the same molar excess. The labeling process is probabilistic. Thus, approximately 50% of the homodimers are labeled with the same Atto dye (either two Atto 550 or two Atto 647N), while the remaining 50% are labeled with both Atto dyes (one Atto 550 and one Atto 647N), assuming a total degree of labeling of 1 (i.e., 100%). Protein species that do not contain a FRET pair were not separated out. The spectrophotometrically determined DOL_dye_ was found to be 0.5 for Atto 550 and 0.4 for Atto 647N, that is, 90% of purified Hsp90 monomers were labeled with dyes in an almost equal dye ratio. From this, the proportion of fully labeled dimeric Hsp90 is derived to be 81% $\left(0.4∙0.5+0.5∙0.4+0.5∙0.5+0.4∙0.4=0.81\right)$ of which 40% are labeled with a FRET pair $\left(0.4∙0.5+0.5∙0.4=0.40\right)$. The labeling efficiency with single‐stranded methyltetrazine‐modified DNA oligonucleotide (DOL_oligo_) was determined to be 71% using an SDS‐PAGE‐based approach (Figure 2d).

### 2.3 Mechanical readout in optical tweezers experiments

**FIGURE 3:**
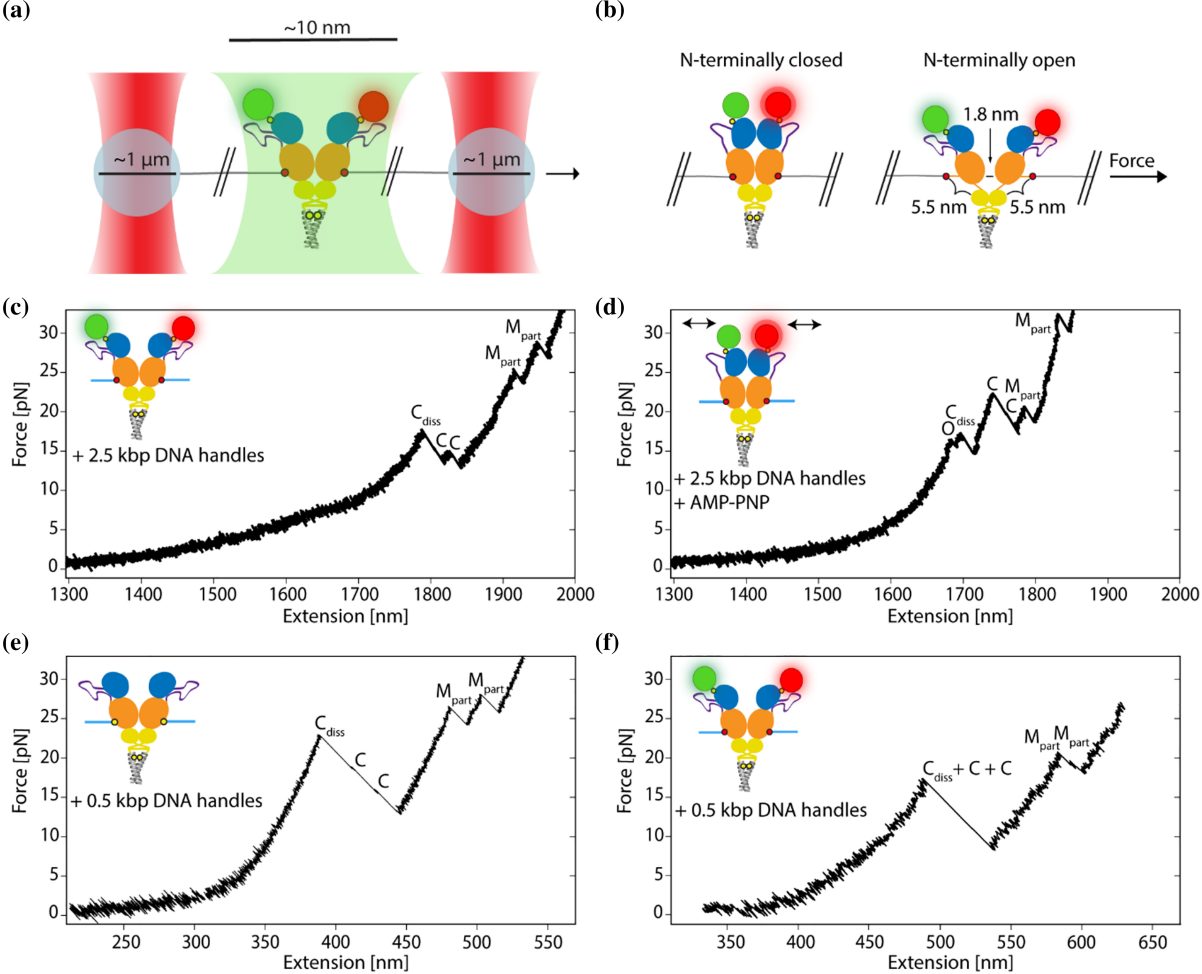
Force readout of fluorescently labeled Hsp90 in optical tweezers experiments. (a) Schematic illustration of the experimental setup: Hsp90 labeled with a FRET pair is tethered between two trapped polystyrene beads using 2.5 kbp‐long DNA handles. Hsp90 domains are displayed in different colors: NTD (blue), linker (purple), MD (orange), CTD (yellow), and the artificial coiled‐coil motif (gray). Introduced non‐canonical amino acid CpK (red circle) and cysteines (yellow circle) with attached fluorescent dyes Atto 550 (green circle) and Atto 647N (red circle). Excitation laser light is depicted in green. (b) Visual aid for length gain between the N‐terminally closed state and N‐terminally open state under applied force. (c) Example unfolding trace of fluorescently labeled Hsp90 using 2.5 kbp‐long DNA handles in a constant velocity (500 nm s^−1^) optical tweezers experiment. The trace shows the initial dissociation of the Hsp90 CTDs (C_diss_), followed by the unfolding of both Hsp90 CTDs (C), and finally the unfolding of both Hsp90 MDs (M_part_). (d) Similar to (c), but using fluorescently labeled Hsp90 in presence of AMP‐PNP, known to promote the formation of the N‐terminally closed state of Hsp90. The trace shows an additional length change resulting from the transition from the N‐terminally closed to the open conformation (O), followed by the dissociation of the Hsp90 CTDs (C_diss_), unfolding of both Hsp90 CTDs (C), and finally the unfolding of both Hsp90 MDs (M_part_). (e) Similar to (c), but using non‐fluorescently labeled Hsp90 and 0.5 kbp‐long DNA handles. (f) Similar to (c), but using 0.5 kbp‐long DNA handles.

The dissociation and unfolding of the Hsp90 dimer under force applied at amino acid position 452 in the MD, using 0.5 kbp handles, have been previously characterized in constant velocity experiments at 500 nm s^−1^ using an optical tweezers setup (Tych et al. 2018). This study observed reproducible changes in contour length, attributed to the dissociation and unfolding of the CTD (amino acids 536–655) and the partial unfolding of the middle domain (MD_part_, amino acids 453–527), by comparing these changes to the Hsp90 crystal structure. In our study, optical tweezers experiments were conducted with the same Hsp90 construct at the same pulling velocity of 500 nm s^−1^, using the non‐canonical amino acid CpK at position 452 instead of cysteine, and with position 61 in the NTD of dimeric Hsp90 labeled with Atto 550 and Atto 647N, a FRET pair (Figure 3a).

For dye‐labeled Hsp90, measurements were conducted using 2.5 kbp handles, both in the absence and presence of the nucleotide AMP‐PNP, a known inducer of the closed state of the N‐terminal domains of the dimeric Hsp90 (Ratzke et al. 2014). Five times longer handles were used for dye‐labeled Hsp90 to increase the distance between the dyes and the high‐intensity trapping lasers to minimize photobleaching of the dyes. This resulted in 147 unfolding traces obtained in the absence of AMP‐PNP and 26 traces in its presence, examples of which are shown in Figure 3c,d. Observed events are comparable to previously published data of the Hsp90 construct without attached dyes (Figure 3e and Table 1), also measured at a velocity of 500 nm s^−1^ but using 0.5 kbp handles instead (Tych et al. 2018). In the presence of AMP‐PNP, an additional observation was made: a reproducible contour length gain of 11.9 nm (±5.6 nm) at a low force of 8.8 pN (±3.8 pN) across various traces (event “O” in Figure 3d). This event has also been observed for non‐dye‐labeled Hsp90 (Figure S1) and consistently occurred before the dissociation and unfolding of the Hsp90 CTDs and the unfolding of the MD_part_ domains, likely due to the opening of the N‐terminally closed dimer. In single‐molecule FRET experiments, the distance between residues at position 452 was found to be 6.1 nm in the closed state and 7.9 nm in the open state of Hsp90 (Hellenkamp et al. 2017), demonstrating an increase of 1.8 nm (7.9–6.1 nm) with N‐terminal dimer opening (Figure 3b). Upon applying force, an unstructured region connecting the Hsp90 MD and CTD, as well as the loop region around the force application point, contributes an additional extension of 5.5 nm per Hsp90 monomer (Figure 3b). Consequently, the total expected increase in length from the closed state of Hsp90 to the open state, with the C‐terminal dimerization interface intact, is estimated at approximately 12.8 nm (5.5 nm × 2 + 1.8 nm). This approximation matches well with the observed contour length gain of 11.9 nm (±5.6 nm).

**TABLE 1:** Length gains and dissociation or unfolding forces from previously published data (Tych et al. 2018) of non‐dye labeled middle‐domain Hsp90 attachment construct measured using 0.5 kbp DNA handles (H in the table below), alongside data of the dye‐labeled construct measured with the same DNA handle length for direct comparison.

Domain, event	Measured length gain (nm) (±SD)				Dissociation or unfolding force (pN) (±SD)			
	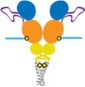	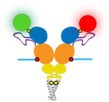	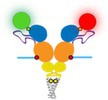	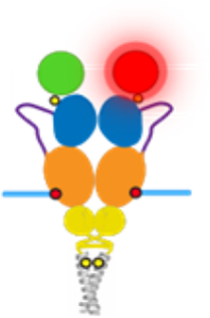	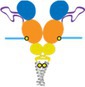	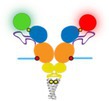	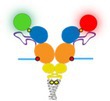	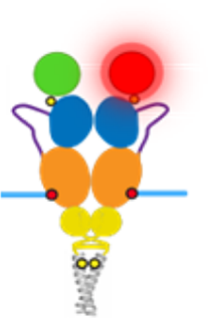
	Non‐dye‐labeled +0.5 kbp H (data from Tych et al. 2018)	Dye‐labeled +0.5 kbp H	Dye‐labeled +2.5 kbp H	Dye‐labeled +2.5 kbp H + AMP‐PNP	Non‐dye‐labeled +0.5 kbp H (data from Tych et al. 2018)	Dye‐labeled +0.5 kbp H	Dye‐labeled +2.5 kbp H	Dye‐labeled +2.5 kbp H + AMP‐PNP
N‐terminal dimer opening	n.a.	n.a.	n.a.	11.9 (±5.6)	n.a.	n.a.	n.a.	8.8 (±3.8)
C‐terminal dimer dissociation	44.4 (±1.3)	43.8 (±4.0)	44.9 (±3.0)	44.2 (±6.0)	21.0 (±4.6)	16.8 (±6.0)	16.5 (±3.7)	10.7 (±4.5)
C‐terminal domain unfolding	37.3 (±2.4)	37.9 (±5.0)	40.3 (±3.6)	42.3 (±5.3)	17.5 (±3.2)	19.7 (±4.1)	16.0 (±2.7)	13.2 (±5.6)
M_part_ domain unfolding	24.5 (±1.1)	25.1 (±5.0)	26.8 (±3.2)	27.7 (±4.0)	27.9 (±2.8)	24.3 (±5.0)	26.5 (±2.9)	26.2 (±5.5)

*Note*: Additionally, data of the dye‐labeled construct measured using 2.5 kbp DNA handles to minimize photobleaching of dyes, both in the absence and presence of AMP‐PNP, are included. All measurements were performed at a constant velocity of 500 nm s^−1^.

Additionally, experiments with dye‐labeled Hsp90, using 0.5 kbp handles and a constant velocity of 500 nm s^−1^ (Figure 3f), were performed to allow for a direct comparison with data from non‐dye‐labeled Hsp90 (Tych et al. 2018), for which the same handle length and pulling velocity were used. Data were obtained from 172 unfolding traces. A two‐tailed Student's t‐test conducted to assess the statistical significance of the observed differences between contour length gains as well as dissociation and unfolding forces (Table 1) between the dye‐labeled and non‐dye‐labeled Hsp90 revealed no significant differences in contour length gains (C‐terminal dimer dissociation: p=0.5, C‐terminal domain unfolding: p=0.2, M_part_ domain unfolding: p=0.5) but significant differences in all forces (C‐terminal dimer dissociation: $p=9.4∙10^{-8}$, C‐terminal domain unfolding: $p=2.0∙10^{-8}$, M_part_ domain unfolding: $p=2.9∙10^{-12}$). While the C‐terminal dimer dissociation force and M_part_ domain unfolding force were significantly lower for the dye‐labeled construct, indicating decreased domain stability, the C‐terminal domain unfolding force was significantly higher, showing increased domain stability compared to the non‐dye‐labeled construct. In summary, the incorporated non‐canonical amino acid, and/or attached dyes do not appear to affect Hsp90's biological function, conformation, or refolding capacity, but the domain stabilities. Scatter plots of data from optical tweezers experiments are displayed in Figure S2.

### 2.4 Fluorescence readout in optical tweezers experiments

**FIGURE 4:**
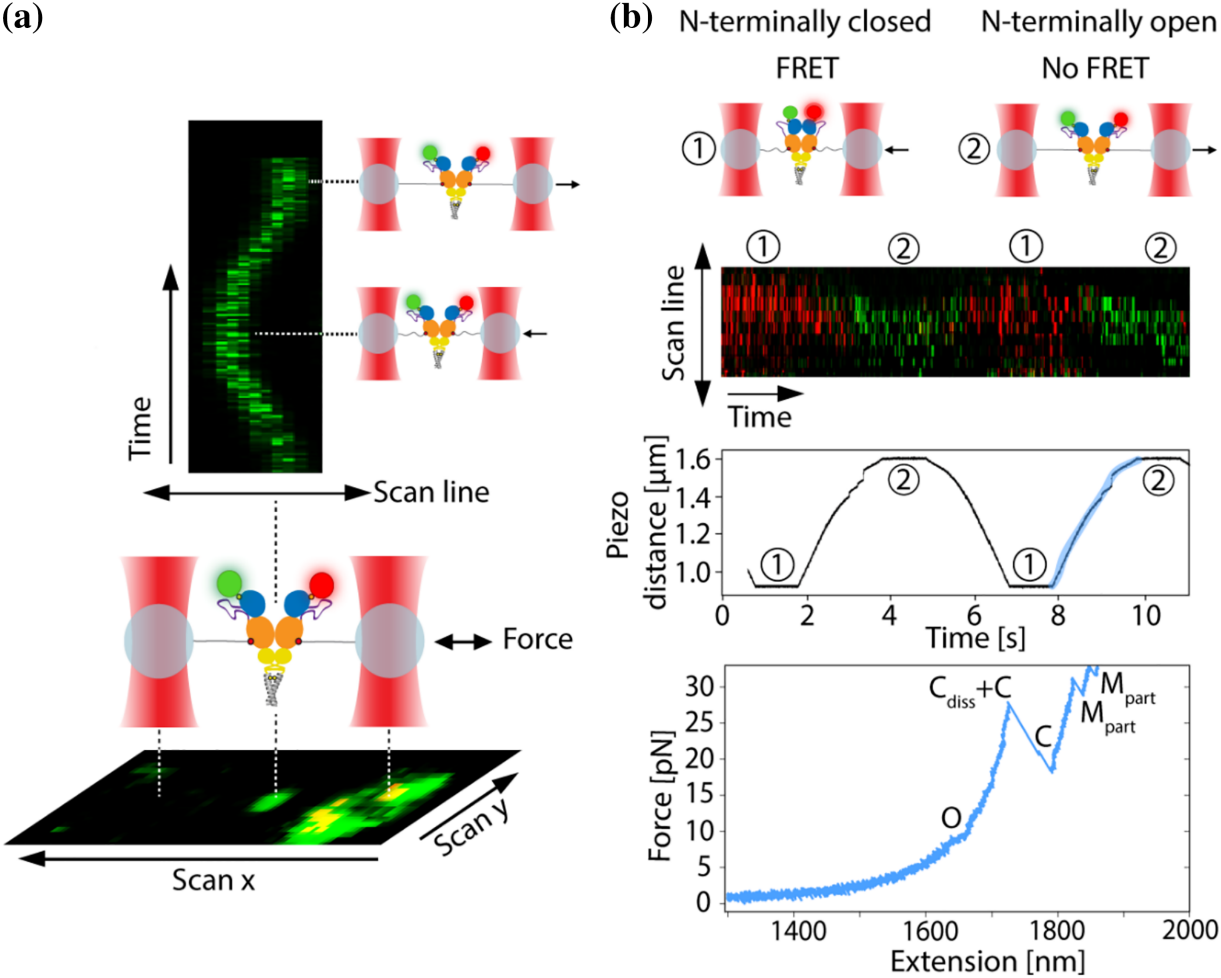
Kymograph recordings. (a) Illustration of an image scan (bottom) and example kymograph recording (top) depicting Hsp90 labeled with the FRET pair Atto 550 (green circle) and Atto 647N (red circle) during repeated stretching and relaxation cycles in the absence of AMP‐PNP. (b) In the N‐terminally closed state of dimeric Hsp90, the fluorescent dyes at positions 61 in the NTD are in close enough proximity for FRET to occur but are too distant in the N‐terminally open state (top panel). The example kymograph recorded in the presence of AMP‐PNP (one panel below top panel) spans two relaxation‐stretching cycles, which can be followed in the piezo distance versus time plot below from the same recording. The plot of piezo distance over time begins at a small piezo distance, corresponding to a relaxed tether that permits the N‐terminal domain of Hsp90 to close, thereby enabling FRET (red signal). As the tether is stretched, the piezo distance progressively increases, resulting in greater force exerted on the protein and inducing a shift to the N‐terminally open conformation of Hsp90 (green signal). In this state, the dyes are separated by a distance too great to allow FRET. The stretching cycle region of the piezo distance graph, corresponding to the displayed unfolding trace (lowest panel), is highlighted in blue. The force‐extension readout indicates the transition from the N‐terminally closed to open conformation (O), followed by the dissociation of the Hsp90 CTDs (C_diss_), unfolding of both CTDs (C), and finally the partial unfolding of both MDs (M_part_). The instrument used had an offset between the green and red channels. To correct this in the displayed kymographs, a custom Python script was used to determine the pixel shift between the two channels. The shift was then applied using Photoshop, which was also used to adjust the brightness for better visualization.

Next to mechanical readout, FRET was used to observe the transition from the N‐terminally closed to open conformation of Hsp90. To enable the detection of fluorescent dyes attached to Hsp90 with the hybrid C‐Trap system, it is crucial to accurately align the confocal focus with the optical trap's focal plane. This procedure is detailed in Figure S3a,b. Additionally, Figure S3c,d presents image scans demonstrating that 2.5 kbp DNA handles enable proper detection of fluorescence from the tethered protein, whereas 0.5 kbp DNA handles do not. To monitor fluorescence along a selected scan line over time during repeated stretching and relaxation cycles, fluorescence data were acquired through kymograph recordings (Figure 4a).

If an excited donor molecule (here, Atto 550) and an acceptor molecule (here, Atto 647N) are separated by less than 10 nm, energy transfer from the donor to the acceptor fluorophore via dipole–dipole interaction (FRET) results in fluorescence of the acceptor (here, red fluorescence, Figure 4b) and simultaneous quenching of the donor fluorescence (here, green fluorescence, Figure 4a,b) (Gust et al. 2014). In the N‐terminally open state of dimeric Hsp90, the two fluorescent dyes at positions 61 in the NTD are too distant for FRET to occur but are in close enough proximity in the N‐terminally closed state (87.8 Å) (Figure 4b) (Hellenkamp et al. 2017). Measurements were therefore also performed in the presence of AMP‐PNP (Figure 4b), which strongly shifts Hsp90 to the N‐terminally closed state. Upon the application of force to Hsp90, a decrease in red fluorescence and an increase in green fluorescence were observed. This indicates a transition from the N‐terminally closed conformation, with the FRET pair in close proximity, to the N‐terminally open conformation with the FRET pair too far apart for FRET to occur. For clarity, Figure 4 shows only the scanned kymograph region of the tethered protein. Kymograph recordings, including the scanned region of the beads, are presented in Figure S4. The kymograph recording in Figure 4b spans two relaxation‐stretching cycles, which can be followed in the piezo distance versus time plot (middle panel in Figure 4b). The piezo distance versus time plot starts at a small piezo distance, corresponding to a relaxed tether, allowing N‐terminal closure of Hsp90 and the occurrence of FRET. The piezo distance then increases as the tether is stretched, leading to an increase in the force applied to the protein and a transition to the N‐terminally open conformation of Hsp90, that is, a state in which the attached dyes are too far apart for FRET to occur. One of the two stretching regions is highlighted in blue in the piezo distance versus time graph. This stretching region corresponds to the unfolding trace shown in the lowest panel of Figure 4b, which includes the mechanical readout of the transition from the N‐terminally closed to the N‐terminally open conformation of Hsp90 (“O” in the lowest panel of Figure 4b).

## 3 DISCUSSION

Optical tweezers have revolutionized the study of biological macromolecules by enabling precise force manipulation at the single‐molecule level. However, their traditional one‐directional probing capability limits the comprehensive characterization of conformational dynamics. These limitations can be overcome by integrating fluorescence microscopy with optical tweezers. In this study, we developed a method to enable the observation of conformational changes and mechanical unfolding of a tethered protein labeled with a FRET pair using a system that has already been well characterized in OT experiments—Hsp90 (Jahn et al. 2014; Jahn et al. 2016; Jahn et al. 2018; Tych et al. 2018).

Central to the success of this integrative approach is the use of selective and efficient orthogonal protein conjugation methods that preserve protein function. In this study, genetic code expansion, coupled with a cysteine mutation, was employed to create specific chemical attachment sites. Previously used strategies for fluorescent labeling include fusing the protein of interest with fluorescent proteins. However, fluorescent proteins are prone to rapid photobleaching, exhibit relatively low fluorescence emission, and are relatively large, potentially causing steric hindrance and interference with the function of the protein of interest (Bustamante et al. 2021; Gross et al. 2010). Therefore, for single‐molecule experiments, organic dyes are generally more suitable. For covalent labeling of proteins with organic dyes and DNA oligonucleotides, different conjugation strategies have already been used. These include peptide sequences such as ybbR (Avellaneda et al. 2020) and LPXTG (Koussa et al. 2014). The ybbR and LPXTG peptide sequences for enzymatic conjugation are relatively short and can be incorporated not only at the N‐ or C‐terminus of a protein but also into solvent‐accessible loops (Guimaraes et al. 2013; Yin et al. 2005). However, an additional enzyme is required to link these short peptide sequences to another peptide or coenzyme for labeling. Enzymatic self‐labeling strategies include the Halo‐tag (Patrick et al. 2020) and SNAP‐tag (Cole 2013), which can be expressed as a fusion protein and do not require the addition of an enzyme. However, these tags are relatively large and can only be added to the N‐ or C‐terminus. The advantage of the labeling strategy used here is that only a single amino acid is required for labeling. Any surface‐accessible amino acid not directly involved in protein function can serve as a potential target for point mutations to introduce chemical attachment sites. Taken together, this approach increases flexibility in selecting a labeling position and minimizes the likelihood of impairing the function of a protein, compared to previously used methods involving the introduction of a peptide tag or the addition of a fusion protein.

An optimized system for incorporating the non‐canonical amino acid CpK resulted in a yield of 1.8 mg of purified protein from a 1 L culture. This amount of protein is sufficient to perform a large number of optical tweezers experiments. Labeling of Hsp90 was achieved in a one‐pot approach using orthogonal chemistries, resulting in a high DOL with both fluorescent dyes, consistent with previous reports (Gebhardt et al. 2021), and single‐stranded DNA oligonucleotides.

The Hsp90 construct labeled in this manner exhibited structural transitions consistent with those observed in a previous optical tweezers study (Tych et al. 2018). The incorporation of the non‐canonical amino acid and the labeling of Hsp90 did not affect the protein's conformation or its refolding ability in OT experiments. Additionally, ATPase activity, used as a measure of the construct's biological function, was comparable to previously reported values for Hsp90_WT_ (Sohmen et al. 2023) making impaired biological function unlikely. However, significant differences in dissociation and unfolding forces between dye‐labeled and non‐dye‐labeled Hsp90 were observed, indicating that the incorporated non‐canonical amino acid and/or attached dyes influence domain stability, potentially by hydrophobic interactions of the fluorescent dyes with the protein (Gust et al. 2014; Zanetti‐Domingues et al. 2013).

The attached FRET pair confirmed the proximity of the labeled residues in the N‐terminally closed state of Hsp90, consistent with existing single‐molecule FRET data (Hellenkamp et al. 2017). This validation not only confirms the experimental approach but also highlights its utility in studying biomolecular conformational transitions.

Expanding this approach to other protein systems that exhibit internal correlated motions along multiple coordinates, such as membrane proteins (van der Sleen et al. 2024; Yang et al. 2018), kinases (Gough and Kalodimos 2024; Xie et al. 2020), and mechanoenzymes (Mukherjee et al. 2023; Rassier & Månsson, 2025), will allow us to provide deeper insights into their complex conformational dynamics. Notably, combining optical tweezers and FRET has already contributed to a better understanding of the coordination between the mechanical and chemical cycles of myosin V (Gunther et al. 2020). Optical tweezers studies examining allosteric communication in proteins (England et al. 2018; Hao et al. 2019; Sonar et al. 2020) can benefit from integrating FRET measurements. This combination enables the direct observation of changes in the relative positions of protein structural elements with high spatial resolution, while simultaneously monitoring the protein's folding energy landscape. Many open questions remain, answers to which this approach could help provide, such as the mechanochemical coupling between ATP hydrolysis and protein unfolding in proteasomal AAA+ motors (Arkinson et al. 2024). Additionally, this labeling approach can be employed to precisely locate protein interaction sites in an optical tweezers setup by attaching one FRET probe to the tethered protein and the other to a protein in solution, allowing for the simultaneous detection of changes in mechanical properties, such as stretching‐induced interactions of proteins involved in mechanotransduction (Del Rio et al. 2009) and the modulation of protein structural stability upon binding an interaction partner (Dahal et al. 2020; Mondol et al. 2023).

## 4 CONCLUSIONS

In conclusion, the integration of fluorescence microscopy with optical tweezers, supported by orthogonal protein labeling strategies, provides a powerful tool for studying conformational dynamics at the single‐molecule level. The use of an *E. coli*‐based protein synthesis system to generate protein constructs, combined with a one‐pot dual labeling approach using commercially available reagents, ensures both efficiency and ease of implementation. By overcoming the limitations of traditional force spectroscopy, this approach opens new avenues for exploring biomolecular systems that exhibit conformational dynamics along multiple coordinates.

## 5 MATERIALS AND METHODS

### 5.1 Expression

Chemically competent *E. coli* K12 underwent co‐transformation with two plasmids: a pBAD plasmid carrying Hsp90 with a D61C mutation and an amber stop codon at D452, and a pEVOL plasmid carrying *Methanosarcina barkeri* (*Mb*) wt pyrrolysyl‐tRNA synthetase and the corresponding *Mb* tRNA.

Following recovery in 1 mL of SOC medium for an hour at 37°C, the cells were cultured overnight in 50 mL of 2 × YT medium supplemented with chloramphenicol (25 μg mL^−1^) and ampicillin (50 μg mL^−1^) at 37°C and 180 rpm. This overnight culture was then diluted to an OD_600_ of 0.05 in 200 mL of fresh 2 × YT medium with the same supplements and incubated at 37°C with agitation at 180 rpm until reaching an OD_600_ of 0.6. Subsequently, 1 mM of CpK (SiChem GmbH, Germany) and 0.02% (w/v) of arabinose were added, inducing protein expression for 16 h at 20°C. Finally, the cells were harvested via centrifugation at 8000*g* for 20 min at 4°C.

### 5.2 Protein preparation

Cells were lysed by sonication in a buffer containing 40 mM HEPES (pH 7.5), 150 mM NaCl, 10 mM imidazole, and 1 mM MgCl_2_ with an EDTA‐free protease inhibitor cocktail added. Protein preparations underwent a multi‐step protocol: first, Ni‐IMAC (Ni‐NTA Agarose, Qiagen, Germany) performed at 4°C to minimize protease activity, followed by size exclusion chromatography (SEC) (Superdex 200, Cytiva, Germany). Fractions of highest purity were combined and incubated with 10 mM TCEP for 30 min at room temperature (21°C) in the dark to reduce cysteines. TCEP removal was achieved via SEC (Superdex 200, Cytiva, Germany), followed by a 1 h room temperature (21°C) incubation for dimerization, covalently linking formed homodimers through a disulfide bond in the artificial coiled‐coil motif. Next, protein in PBS (pH 6.7) buffer was labeled in a one‐pot approach with a 3‐fold molar excess of an equimolar mix of maleimide‐Atto 550 and maleimide‐Atto 647N FRET pair (ATTO‐TEC, Germany) along with a 3‐fold molar excess of single‐stranded methyltetrazine‐modified DNA oligonucleotide (Biomers, Germany) for 2 h at room temperature (21°C) in the dark. The maleimide group, reactive toward solvent‐exposed cysteines, enabled dye attachment at position 61. DNA oligonucleotides were attached at position 452 via IEDDAC of methyltetrazine with the non‐canonical amino acid CpK. Finally, SEC was used to remove free oligos, dyes, and aggregated protein, and to exchange the buffer to 40 mM HEPES (pH 7.5), 150 mM KCl, and 10% glycerol.

### 5.3 Activity assay

ATPase activity was measured at 37°C using an assay similar to that described by Tamura and Gellert (1990), which couples the hydrolysis of ATP to NADH oxidation. The following mix of reagents was used: 0.2 mM NADH, 3 mM phosphoenolpyruvate potassium salt, 13 U mL^−1^ lactate dehydrogenase, and 3 U mL^−1^ pyruvate kinase in a buffer containing 40 mM HEPES (pH 7.5), 150 mM KCl, and 10 mM MgCl_2_. After the addition of 2 μM Hsp90, the linear decrease in the concentration of NADH, which absorbs at 340 nm, was monitored spectrophotometrically for 15 min. 50 μM radicicol was added to inhibit the reaction and measure the background ATPase activity for 5 min.

### 5.4 Determination of the average degree of labeling

DOL_dye_, which refers to the average number of dye molecules attached to each protein molecule, was determined for the conjugate solution obtained after the final SEC step. This was accomplished using absorption spectroscopy and applying the Lambert–Beer law using the theoretical extinction coefficient of the monomeric Hsp90 construct (*ε*_prot_ = 75,415 M^−1^ cm^−1^). Absorbance measurements were taken at the dyes' absorption maxima (*A*_max_) as well as at the protein's absorption wavelength, 280 nm (*A*_prot_). Since all dyes exhibit absorbance at 280 nm, the measured absorbance at this wavelength (*A*_280_) was corrected to account for the dyes' contribution. This correction followed the method outlined by the dye supplier ATTO‐TEC, utilizing the provided correction factors (${\mathrm{CF}}_{280}$) and dye extinction coefficients ($\varepsilon_{\max}$),

$$\begin{array}{rcl} {\mathrm{DOL}}_{\mathrm{dye}} & = & \frac{c\left(\mathrm{dye}\right)}{c\text{(protein)}}=\frac{A_{\max}/\varepsilon_{\max}}{A_{\text{prot}}/\varepsilon_{\text{prot}}}\\ & = & \frac{A_{\max}∙\varepsilon_{\text{prot}}}{\left(A_{280}-A_{\max}∙{\mathrm{CF}}_{280}\right)∙\varepsilon_{\max}}. \end{array}$$

DOL_oligo_ was determined by quantifying the bands of oligonucleotide‐labeled and non‐oligonucleotide‐labeled proteins separated on SDS‐PAGE gels using ImageJ.

### 5.5 Attachment of DNA handles

The DNA oligonucleotides bind to the sticky ends of 2.5 kbp (~1.7 μm) DNA handles, functionalized with digoxigenin or biotin. These handles were synthesized in‐house using Lambda DNA as a template (New England Biolabs) and custom‐designed primers (Metabion, Germany). This was done similarly to the synthesis of shorter 0.5 kbp handles used for the non‐fluorescently labeled construct, which is described in Tych and Rief (2022). DNA handles were incubated with oligo‐protein in the presence of 2 mM MgCl_2_ for 1 h at room temperature (21°C) and 150 rpm on a plate shaker.

### 5.6 DNA sequences

*DNA oligonucleotide modified with a methyltetrazine group*

5′‐G GCA GGG CTG ACG TTC AAC CAG ACC AGC GAG TCG‐methyltetrazine‐3′.

*Custom‐designed primers are used to make DNA handles*

5′‐Biotin‐GTAC(Biotin‐dT)GGA(Biotin‐dT)GCACTG GAG AAG‐3′.

5′‐Digoxigenin‐GTAC(Dig‐dT)GGA(Dig‐dT)GCACTG GAG AAG‐3′.

5′‐CGACTCGCTGGTCTGGTTGAACGTCAGCCCTGCC(abasic site)CCTGCCCGGCTCTGGACAGG‐3′.

### 5.7 Optical tweezers experiments

Optical tweezers experiments using a C‐trap instrument (Lumicks, the Netherlands) that combines a confocal microscope with optical tweezers were performed as described before (Mondol et al. 2023), but using 0.75 μm antidigoxigenin and 1.36 μm streptavidin‐coated polystyrene beads and conducting the measurements at a constant velocity of 500 nm s^−1^ instead. In addition to the used oxygen scavenger system—comprising 1700 U mL^−1^ glucose catalase, 27 U mL^−1^ glucose oxidase, and 0.66% glucose to minimize photodamage caused by oxygen free radicals—1 mM Trolox was included in the measurement buffer of dye‐labeled constructs as an antiblinking and antibleaching reagent. The addition of Trolox has been shown not to affect the unfolding pattern or domain stability of Hsp90 (Jahn et al. 2014; Jahn et al. 2016).

To align the confocal focus (determined by the objective position) with the trap focal plane, where the beads are trapped with the tethered sample, SYBR Safe stain (Invitrogen) was employed at a final concentration of 250× (achieved by diluting the 10,000× stock solution 40 times).

Measurements with fluorescently labeled Hsp90 construct were additionally performed in the presence of 2 mM AMP‐PNP, following incubation with the sample for 30 min at room temperature (21°C). Fluorescence data were collected using the hybrid Lumicks instrument's confocal system, which is equipped with three excitation lasers (488 nm, 532 nm, 638 nm) and three single‐photon‐sensitive avalanche photodiodes with bandpass filters (APDs, APD1: 500–550 nm, APD2: 575–625 nm, APD3: 635–835 nm). For data acquisition, a 532‐nm excitation laser was used at 10% power, along with the kymograph function of the software used to run the Lumicks C‐trap, Bluelake. Fluorescence was detected using avalanche photodiodes APD2 and APD3. The kymograph settings included a pixel size of 100 nm, a pixel dwell time of 0.3 ms, and a line scan time of 11.9 ms. Analysis of force data was done as described before (Mondol et al. 2023), but with an expected DNA contour length of 1735 nm for 2.5 kbp‐long DNA handles.

## AUTHOR CONTRIBUTIONS

**Laura‐Marie Silbermann:** Conceptualization; methodology; validation; writing – original draft; investigation; formal analysis; visualization; writing – review and editing; data curation. **Maximilian Fottner:** Writing – review and editing; methodology. **Ronald van der Meulen:** Formal analysis; data curation; validation; investigation. **Nora Migdad:** Investigation; writing – review and editing. **Kathrin Lang:** Resources; supervision. **Katarzyna Tych:** Conceptualization; investigation; funding acquisition; writing – review and editing; project administration; supervision; resources; data curation; software.

## CONFLICT OF INTEREST STATEMENT

The authors declare no conflicts of interest.

## Supporting information


**Data S1.** Supporting Information.

## Data Availability

The data that support the findings of this study are openly available in Mendeley Data at https://data.mendeley.com/datasets/2hyfhfnrnp/1, reference number DOI: https://doi.org/10.17632/2hyfhfnrnp.1.
